# Neutrophil Immunomodulatory Activity of Farnesene, a Component of *Artemisia* *dracunculus* Essential Oils

**DOI:** 10.3390/ph15050642

**Published:** 2022-05-23

**Authors:** Igor A. Schepetkin, Gulmira Özek, Temel Özek, Liliya N. Kirpotina, Andrei I. Khlebnikov, Robyn A. Klein, Mark T. Quinn

**Affiliations:** 1 Department of Microbiology and Cell Biology, Montana State University, Bozeman, MT 59717, USA; igor@montana.edu (I.A.S.); liliya@montana.edu (L.N.K.); 2 Department of Pharmacognosy, Faculty of Pharmacy, Anadolu University, Eskisehir 26470, Turkey; gulmiraozek@gmail.com (G.Ö.); temelozek@gmail.com (T.Ö.); 3 Kizhner Research Center, Tomsk Polytechnic University, 634050 Tomsk, Russia; aikhl@chem.org.ru; 4 Department of Plant Sciences and Plant Pathology, Montana State University, Bozeman, MT 59717, USA; herbrobin@gmail.com

**Keywords:** anti-inflammatory, *Artemisia*, calcium flux, chemotaxis, essential oils, farnesene, monoterpene, neutrophil

## Abstract

Despite their reported therapeutic properties, not much is known about the immunomodulatory activity of essential oils present in *Artemisia* species. We isolated essential oils from the flowers and leaves of five *Artemisia* species: *A. tridentata*, *A. ludoviciana*, *A. dracunculus*, *A. frigida*, and *A. cana*. The chemical composition of the *Artemisia* essential oil samples had similarities and differences as compared to those previously reported in the literature. The main components of essential oils obtained from *A. tridentata*, *A. ludoviciana*, *A. frigida*, and *A. cana* were camphor (23.0–51.3%), 1,8-cineole (5.7–30.0%), camphene (1.6–7.7%), borneol (2.3–14.6%), artemisiole (1.2–7.5%), terpinen-4-ol (2.0–6.9%), α-pinene (0.8–3.9%), and santolinatriene (0.7–3.5%). Essential oils from *A. dracunculus* were enriched in methyl chavicol (38.8–42.9%), methyl eugenol (26.1–26.4%), terpinolene (5.5–8.8%), (*E*/*Z*)-β-ocimene (7.3–16.0%), β-phellandrene (1.3–2.2%), *p*-cymen-8-ol (0.9–2.3%), and xanthoxylin (1.2–2.2%). A comparison across species also demonstrated that some compounds were present in only one *Artemisia* species. Although *Artemisia* essential oils were weak activators of human neutrophils, they were relatively more potent in inhibiting subsequent neutrophil Ca^2+^ mobilization with *N*-formyl peptide receptor 1 (FPR1) agonist *f*MLF- and FPR2 agonist WKYMVM, with the most potent being essential oils from *A. dracunculus*. Further analysis of unique compounds found in *A. dracunculus* showed that farnesene, a compound with a similar hydrocarbon structure as lipoxin A_4_, inhibited Ca^2+^ influx induced in human neutrophils by *f*MLF (IC_50_ = 1.2 μM), WKYMVM (IC_50_ = 1.4 μM), or interleukin 8 (IC_50_ = 2.6 μM). Pretreatment with *A. dracunculus* essential oils and farnesene also inhibited human neutrophil chemotaxis induced by *f*MLF, suggesting these treatments down-regulated human neutrophil responses to inflammatory chemoattractants. Thus, our studies have identified farnesene as a potential anti-inflammatory modulator of human neutrophils.

## 1. Introduction

The genus *Artemisia* is one of the most broadly distributed groups in the Asteraceae family [1]. Within this family, *Artemisia* is in the tribe Anthemideae and comprises about 530 species of plants that are found on all continents except for Antarctica. These plants are found mainly in the Northern Hemisphere, with only 25 species in the Southern Hemisphere [2]. According to the latest taxonomic revisions, there are 50 *Artemisia* species in North America, which belong to *Artemisia* (Miller) Less, *Absinthium* (Miller) Less, *Dracunculus* Besser, and *Tridentatae* (Rydb.) subgenera [3]. *Artemisia* is most prevalent in the western half of the United States, with 11 species in or near the Rocky Mountain region [4]. According to the United States Department of Agriculture, 15 *Artemisia* species have been described: *A. arbuscula* Nutt.,*A. bigelovii* Gray, *A. californica* Less., *A. cana* Pursh., *A. filifolia* Torr., *A. frigida* Willd., *A. longiloba* (Osterhout) Beetle, *A. lugoviciana* Nutt., *A. nova* A.Nels., *A. pygmaea* Gray, *A. rigida* (Nutt.) Gray, *A. rothrockii* Gray, *A. spinescens* Eaton, *A. tridentata* Nutt., and *A. tripartita* Rydb. [5].

The importance of *Artemisia* species in traditional medicine is evident in a number of ethnopharmacological reports [6,7,8]. Asteraceae-specific ethnobotanical reports recorded in the Native American Ethnobotany database revealed *Artemisia* species as the second most selected medicinal taxa after *Achillea*. *A. tridentata* was most commonly cited as a pulmonary aid, whereas *A. dracunculus* L., *A. tridentata*, and *A. douglasiana* Besser were cited for orthopedic uses [9]. Many *Artemisia* species are characterized by a strong odor due to essential oils that are mainly concentrated in their leaves and flowers. The pharmacological properties of *Artemisia* essential oils have been reported, including antibacterial, antiparasitic, antinociceptive, hypoglycemic, and antioxidant activities [10,11,12,13,14,15,16,17].

Essential oils from various plant species have been reported to exhibit immunomodulatory activity. For example, *Ocimum kilimandscharicum* Gürke leaf essential oils inhibited carrageenan-induced leukocyte rolling, adhesion, and *f*MLF-induced leukocyte chemotaxis [18]. Likewise, *Juniperus* essential oils inhibited the production of the proinflammatory cytokines tumor necrosis factor (TNF), interleukin (IL)-1β, and γ-interferon by lipopolysaccharide-stimulated leukocytes [19]. Recently, we found that essential oils from *Artemisia kotuchovii*, *Ferula akitschkensis*, *F. iliensis*, *Hypericum perforatum*, *Rhododendron albiflorum*, and *Juniperus* spp. can activate or inhibit human neutrophil function [20,21,22,23,24,25].

Here, we characterized the chemical composition and immunomodulatory activity of the flower and leaf essential oils from five *Artemisia* species collected in Montana and analyzed their chemical compositions and innate immunomodulatory activities. We show that several of the *Artemisia* essential oils potently inhibited intracellular Ca^2+^ mobilization [Ca^2+^]_i_ in human neutrophils, with the most active being essential oils from *A. dracunculus*. Furthermore, we demonstrated farnesene, a unique component of *A. dracunculus*, inhibited human neutrophil activation and chemotaxis and is likely one of the main active components. Based on the critical role of neutrophils in inflammation, our data support the possibility that farnesene could have the potential for the development of new anti-inflammatory agents.

## 2. Results and Discussion

### 2.1. Essential Oil Composition

Essential oils were obtained from the flowers (designated by Fl) and leaves (designated by Lv) of five *Artemisia* species for subsequent phytochemical and biological characterization. Plant material was collected from wild plants in 2019 around Bozeman and Three Forks (MT, USA) (Table 1). After botanical identification of the plant material, the flowers and leaves were dried for 7–10 days at room temperature but protected from direct sunlight.

The yields (*v*/*w*) of *Artemisia* spp. essential oils ranged from 0.3 to 3.3% (Table 1). The composition of the extracted essential oils was determined using simultaneous GC-FID and GC/MS. Twenty-seven major compounds found in the essential oils are shown in Table 2 and Supplementary Table S1 shows the total of all compounds identified in these samples. The main classes of compounds in all *Artemisia* species were oxygenated monoterpenes, ranging from 1.9 (AD_Fl_) to 84.8% (AC_Lv_), and monoterpene hydrocarbons, which ranged from 6.5 (AC_Lv_) to 25.8% (AD_Fl_). Essential oils from *A. tridentata*, *A. ludoviciana*, *A. frigida*, and *A. cana* contained high amounts of oxygenated monoterpenes (77.4–84.8%), with 1,8-cineole and camphor being the most representative, which is consistent with previous reports for *A. ludoviciana*, *A. frigida*, and *A. cana* essential oils [26]. In addition, essential oils from the *A. dracunculus* were enriched in phenylpropanoids (65.3–69.0%), including methyl chavicol (estragole) and methyl eugenol, also supporting previous reports analyzing essential oils from aerial parts of this plant [26,27]. Note that this is the first report comparing individual leaf and flower essential oils from these *Artemisia* species.

*Artemisia* exhibits significant intraspecies variations in terpenes present in their essential oils [16,26,34,35,36,37,38,39,40,41,42,43,44,45,46,47,48,49,50,51,52,53,54,55,56,57,58]. For example, the variation in volatile components of these plants may occur during plant maturation at different altitudes. Our studies confirmed these findings and extended this analysis by providing the first report comparing essential oils isolated from preparations of *A. tridentata* flowers and leaves prepared from the same plant samples. Interestingly, we found that some compounds exhibited remarkable differences in terms of their presence in different parts of the same *Artemisia* species. For example, santolinatriene and artemiseole were found only in the flowers but not in the leaves of *A. ludoviciana*. Likewise, santolina epoxide and the monoterpene alcohol grandisol, a pheromone, were detected only in *A. cana* flowers. Similarly, *trans*-chrysanthenol and its ester *trans*-chrysanthenyl acetate were detected only in *A. frigida* leaves, and fragranol was found only in *A. cana* leaf essential oils and not those isolated from flowers. Therefore, it is important to evaluate different parts of plants in terms of target compounds. A comparison across species also demonstrated some differences in the presence of compounds, with some compounds (e.g., (*Z*)-β-ocimene, methyl chavicol, (*E*,*E*)-α-farnesene, xanthoxylin), being present in only one *Artemisia* species. It is also interesting that *A. dracunculus* essential oils had more unique compounds that were not present in essential oils from the other *Artemisia* species.

### 2.2. Effect of Artemisia Essential Oils and Pure Compounds on Neutrophil Ca^2+^ Influx

Neutrophils are the most common leukocyte in human blood and are required for innate immune responses [59]. These cells respond rapidly to infection and injury in various tissues. They represent the first line of defense and utilize multiple mechanisms of oxygen-dependent and oxygen-independent processes to protect against infection, including phagocytosis, cytokine secretion, and reactive oxygen species production [60,61]. Thus, neutrophils represent a potential target for the development of novel anti-inflammatory therapeutics. Indeed, natural products, such as essential oils, have exhibited neutrophil immunomodulatory activity [20,21,22,23,24].

*Artemisia* essential oils were evaluated for their immunomodulatory effects on human neutrophils. Initially, we measured the effects of the essential oils on [Ca^2+^]_i_, which is a key component of neutrophil activation and function [62]. We found that treatment of neutrophils with *Artemisia* essential oils only modestly enhanced [Ca^2+^]_i_ at relatively high concentrations, with EC_50_ values ranging from in the range of 15.8 µg/mL (AL_Lv_) to 48.7 µg/mL (AC_Lv_). We also evaluated some of the individual compounds present in the various *Artemisia* essential oils to determine if they were responsible for neutrophil activation, including 1,8-cineole, (+)-camphor, (−)-camphor, (±)-bornyl acetate, farnesene, piperitone, and xanthoxylin. As shown in Table 3, 1,8-cineole, (+)-camphor, (−)-camphor, and piperitone did not affect human neutrophil Ca^2+^ flux, whereas farnesene, xanthoxylin, and (±)-bornyl acetate were weak activators, but only at relatively high concentrations. Note that previous analysis of some of the other major compounds that we also found here to be present in *Artemisia* essential oils (i.e., α-pinene, camphene, (*E*/*Z*)-β-ocimene, *p*-cymene, terpinolene, linalool, terpinen-4-ol, methyl chavicol, *p*-cymen-8-ol, and methyl eugenol) showed that they had no effect on human neutrophil [Ca^2+^]_i_ [24,25].

**Figure 1:**
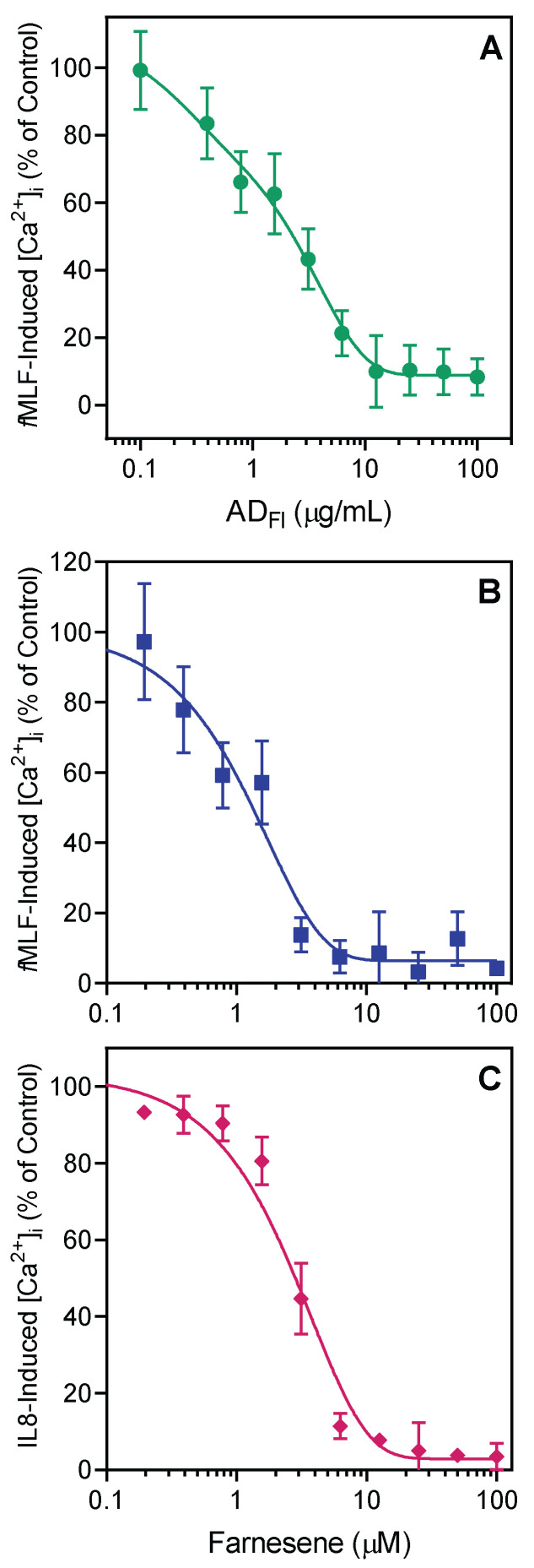
Effect of *A. dracunculus* flower essential oils (AD_Fl_) and farnesene on *f*MLF- and IL-8-induced neutrophil [Ca^2+^]_i_. Human neutrophils were treated for 10 min with AD_Fl_, farnesene, or 1% DMSO (negative control). The cells were then activated with 5 nM *f*MLF (**A**,**B**) or 25 nM of human IL-8 (**C**), and [Ca^2+^]_i_ was monitored as described. The data are shown as the mean ± SD from one experiment. Representative of three (for *f*MLF) or two (for IL-8) independent experiments with similar results.

Since *Artemisia* essential oils and individual compound components exhibited only weak neutrophil activation, we considered whether they might have some effect on neutrophils activated by strong agonists, such as *N*-formyl peptide receptor 1 (FPR1) agonist *f*MLF and FPR2 agonist WKYMVM, as ligands can downregulate or alter neutrophil responses to subsequent treatment with heterologous or homologous agonists [63]. As shown in Table 3, *Artemisia* essential oils were relatively more potent inhibitors of [Ca^2+^]_i_ in *f*MLF- and WKYMVM-stimulated neutrophils, with some IC_50_ values in the low micromolar range. Essential oils from *A. dracunculus* flowers (AD_Fl_) were the most potent, with IC_50_ values of 2.2 and 2.9 μM for inhibition of *f*MLF- and WKYMVM-induced neutrophil [Ca^2+^]_i_, respectively. Representative data showing dose-dependent inhibition of *f*MLF-induced neutrophil [Ca^2+^]_i_, by AD_Fl_ are shown in Figure 1A.

We also evaluated the effect of seven compounds present in *Artemisia* essential oils on *f*MLF- and WKYMVM-induced neutrophil [Ca^2+^]_i_ and found that farnesene, xanthoxylin, and (±)-bornyl acetate inhibited *f*MLF- and WKYMVM-stimulated neutrophils, while the other compounds were inactive (Table 3). Xanthoxylin and (±)-bornyl acetate were also quite weak inhibitors, whereas the most potent inhibitor of peptide-induced neutrophil [Ca^2+^]_i_ was farnesene (IC_50_ = 1.1 ± 0.2 and 1.4 ± 0.5 μM for *f*MLF- and WKYMVM-activated neutrophils, respectively). Representative data showing dose-dependent inhibition of *f*MLF-induced neutrophil [Ca^2+^]_i_, by farnesene are shown in Figure 1B. Likewise, farnesene also dose-dependently inhibited IL-8 induced neutrophil [Ca^2+^]_i_, with an IC_50_ = 2.6 ± 0.2 μM (Figure 1C). These data suggest that farnesene can modulate intracellular signaling pathways that are common to different G protein-coupled receptors (GPCRs), including FPR1/FPR2 and CXCR1/2 chemokine receptors resulting in down-regulation of the response to these chemoattractants.

### 2.3. Effect of A. dracunculus Essential Oils and Pure Compounds on Neutrophil Chemotaxis

**Figure 2:**
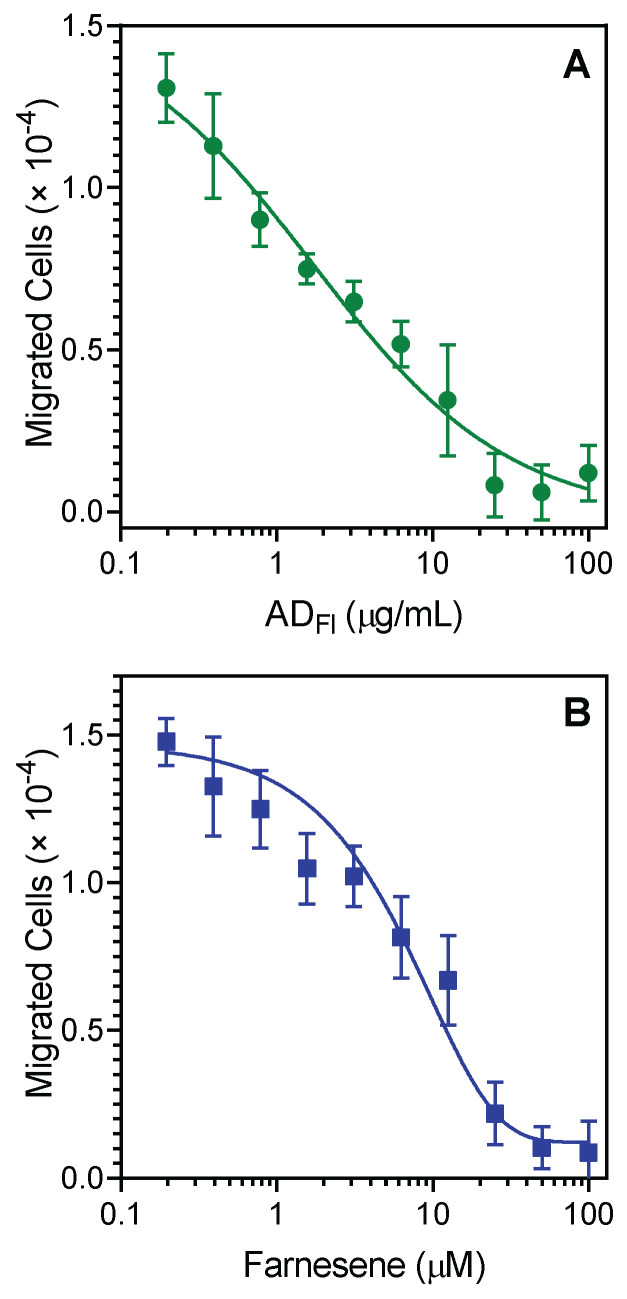
Inhibition of neutrophil chemotaxis by essential oils from *A. dracunculus* flowers (AD_Fl_) (**A**) and farnesene (**B**). Neutrophil chemotaxis toward 1 nM *f*MLF was measured. The data are from one experiment that is representative of two independent experiments.

Since *A. dracunculus* essential oils and their unique component, farnesene, were effective inhibitors of agonist-induced neutrophil [Ca^2+^]_i_, we evaluated their effects on neutrophil chemotaxis. Pretreatment of neutrophils with AD_Fl_ for 30 min dose-dependently inhibited *f*MLF-induced human neutrophil chemotaxis toward *f*MLF, with an IC_50_ of 2.4 ± 1.1 µg/mL (Figure 2A). Likewise, pretreatment of neutrophils with farnesene also effectively inhibited neutrophil migration toward *f*MLF, with an IC_50_ of 3.3 ± 1.4 μM. A representative dose-dependent response for the inhibition of *f*MLF-induced chemotaxis in human neutrophils by farnesene is shown in Figure 2B. Analysis of (±)-bornyl acetate showed that this compound also inhibited *f*MLF-induced neutrophil chemotaxis, although with much lower potency (IC_50_ = 11.9 ± 1.9 μM). In contrast, xanthoxylin did not affect this response.

**Figure 3:**
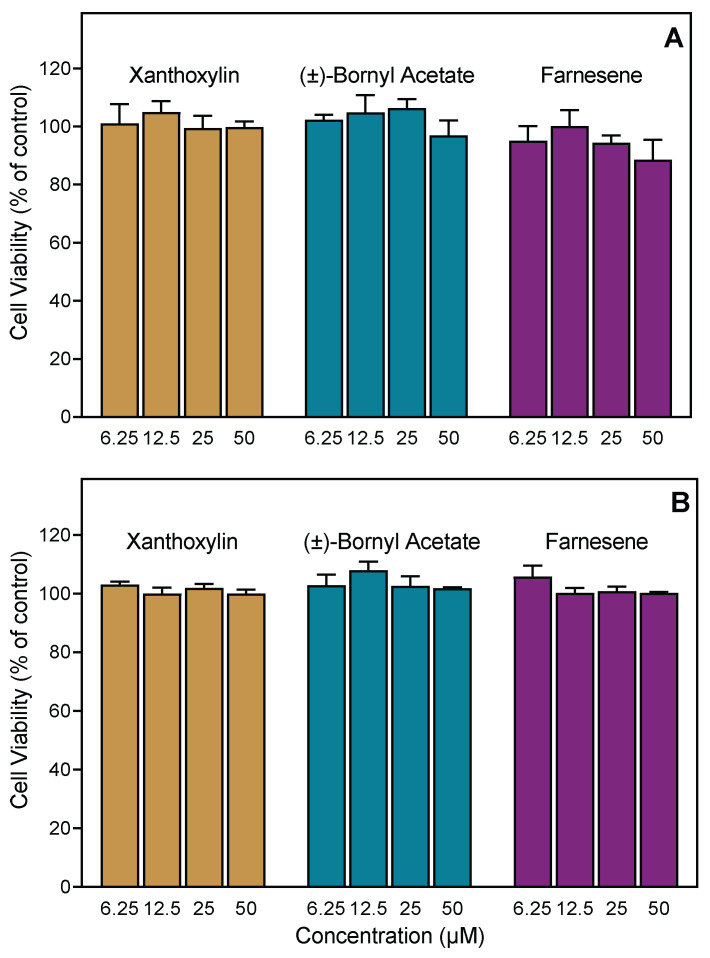
Cytotoxicity of selected compounds. Human neutrophils (**A**) or human THP-1 cells (**B**) were incubated with indicated compounds for 90 min (**A**) or 24 h (**B**), and cell viability was analyzed. The data presented are the mean ± SD of triplicate samples from one experiment. Representative of two independent experiments with similar results.

To ensure that *Artemisia* essential oils or active pure compounds were not cytotoxic, we evaluated the cytotoxicity of the essential oil samples (up to 55 µg/mL) and test compounds at various concentrations (up to 50 µM) in human neutrophils during a 1.5 h incubation period, which covers the times used to measure Ca^2+^ influx (up to 30 min) and cell migration (up to 1.5 h). As shown in Table 3, essential oils from *A. tridentata*, *A. ludoviciana*, and *A. cana* had no cytotoxicity in neutrophils, although essential oils from *A. dracunculus* and *A. frigida* had a little cytotoxicity at the highest concentrations (25–50 μM), which were >10-fold higher than those required to induce biological effects. The pure compounds (±)-bornyl acetate, farnesene, and xanthoxylin were nontoxic in human neutrophils during the 90 min incubation. In addition, these compounds did not affect the viability of human THP-1 monocytic cells after a 24 h incubation period (Figure 3). Thus, the biological effects of these compounds on neutrophil function are not due to compound cytotoxicity and support the conclusion that farnesene is a novel innate immunomodulator.

### 2.4. Identification of Potential Protein Targets for Selected Compounds

Both farnesene and bornyl acetate inhibited neutrophil [Ca^2+^]_i_ and chemotaxis, although farnesene was the most effective by far. Farnesene encompasses a set of six closely related sesquiterpenes, as the α-form can exist as four stereoisomers, and the β-isomer exists as two stereoisomers. We identified both (*Z*,*E*)-α-farnesene and (*E*,*E*)-α-farnesene in *A. dracunculus*, although (*E*,*E*)-α-farnesene was present at a higher level (Table 2). Both α- and β- forms of farnesene have been reported previously in essential oils from *Artemisia* spp. For example, (*Z*,*E*)-α-farnesene was found in *A. maderaspatana* L. [64] and (*E*,*E*)-α-farnesene was present in *A. ordosica* Krasch. and *A. dracunculus* [26,65,66]. (*E*)-β-farnesene was reported to be present in *A. annua* L. [67], *A. magellanica* Sch. Bip. [68], *A. absinthium* L. [69], *A. lavandulifolia* DC. [70], and *A. biennis* Willd. essential oils [26]. Finally, (*Z*)-β-farnesene was found in essential oils from *A. absinthium* [26], *A. annua* [71], and *A. tournefortiana* Reichb. [72]. Although the α- and β-forms of farnesene are well-known pheromones [73,74], we did not find any reports on the biological activity of these compounds in human cells.

Bornyl acetate has been reported previously to be a major component of essential oils isolated from *Artemisia* spp, including *A. ludoviciana* and *A. frigida* [17,53]. Bornyl acetate is also a volatile constituent present in numerous conifer essential oils and essential oils from *Valeriana officinalis* [75]. Previous studies have reported that bornyl acetate has anti-inflammatory properties in different experimental models. For example, bornyl acetate downregulated the levels of proinflammatory cytokines in vitro and in vivo and reduced the number of total cells, neutrophils, and macrophages in murine bronchoalveolar lavage fluid after intranasally injected lipopolysaccharide. Moreover, bornyl acetate inhibited the expression and production of IL-1β and TNF in human umbilical vein endothelial cells (HUVECs) and RAW 264.7 macrophages [76,77]. Bornyl acetate has also been reported to have analgesic effects [78].

To better understand the properties of these compounds, we calculated their most important physicochemical parameters using SwissADME [79]. The LogP values estimated using ALOGPS 2.1 program [80] and tPSA values allowed us to predict that (−) bornyl acetate, (+) bornyl acetate, (*Z*,*E*)-α-farnesene, and (*E*,*E*)-α-farnesene can all permeate the blood–brain barrier (BBB), according to the binary classification tree model [81] (Table 4).

**Figure 4:**
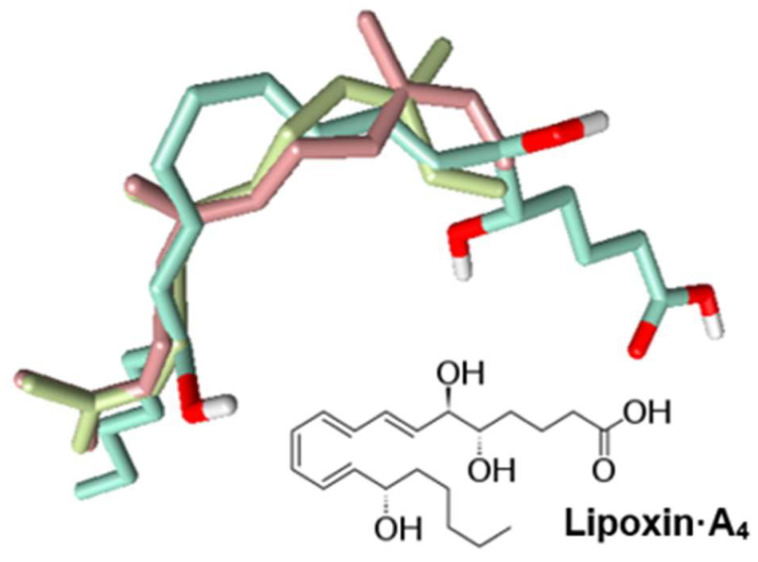
Chemical structure of lipoxin A_4_ and alignment of lipoxin-A_4_ (green), (*E*,*E*)-α-farnesene (pink), and (*Z*,*E*)-α-farnesene (khaki).

Farnesene is very lipophilic (Table 4). Thus, we propose that the neutrophil signaling inhibitory mechanisms may be based on the allosteric interaction of the farnesene chain with the membrane portion of the receptor, and we are currently investigating this mechanism. Indeed, other lipophilic compounds, such as the bile acids deoxycholic acid and chenodeoxycholic acid, have been reported to antagonize FPR1 at high concentrations (>100 µM) [82,83,84]. Moreover, lipoxin A_4_ was also reported as an allosteric modulator of a CB1 cannabinoid receptor and FPR2 [85,86]. To further investigate this issue, we aligned (*E*,*E*)-α-farnesene, (*Z*,*E*)-α-farnesene, and lipoxin A_4_ using FieldTemplater software (Figure 4) and found that the alignment was determined mainly by the hydrophobic hydrocarbon skeletons of the compounds. The combined similarity measure of the superimposition was relatively high (S = 0.690), suggesting that the farnesene molecules could mimic lipoxin A_4_ and maybe other related specialized pro-resolving mediators (known as SPMs), including resolvins, maresins, and protectins [87]. Indeed, many of these molecules have been demonstrated to act allosterically on a number of GPCRs (reviewed in [88]). Interestingly, we found previously that 6-methyl-3,5-heptadien-2-one (MHDO), a compound structurally similar to farnesene, also inhibited neutrophil activity [25], although its molecular targets were not identified.

Not much is known about the specific cellular targets of farnesene or bornyl acetate. Thus, we performed reverse-pharmacophore mapping on the molecular structures of (*Z*,*E*)-α-farnesene, (*E*,*E*)-α-farnesene, and the (−) and (+) forms of bornyl acetate to identify potential biological targets. PharmMapper compared a database of pharmacophore patterns with these compounds and generated target information, such as pharmacophoric characteristics and normalized fitness scores. Note, however, that PharmMapper depends on the availability of structures for pharmacophore mapping, and most GPCRs are not represented. The proper optical isomers of the compounds were submitted to the PharmMapper server as mapping explicitly accounts for the three-dimensional structure of a molecule. The ten top-ranked potential targets found by PharmMapper are shown in Table 5.

PharmMapper analysis indicated that kinases could be among the potential targets for bornyl acetate and farnesene. Among the top ten ranked targets for the (±)-enantiomers of bornyl acetate were c-Jun *N*-terminal kinase 1 (JNK1), 3-phosphoinositide-dependent protein kinase 1 (PDPK1), and transforming growth factor-β receptor type-1 (TGFBR1), a serine/threonine-protein kinase. Likewise, JNK1, p38α mitogen-activated protein kinase (MAPK), extracellular signal-regulated kinase 2 (ERK2), and proto-oncogene serine/threonine-protein kinase (Pim-1) were kinases identified among the ten top-ranked targets for (*E*,*E*)- and (*Z*,*E*)-forms of α-farnesene (Table 5). MAPK signaling plays an important role in neutrophil signal transduction cascades [89], and studies have shown that members of the MAPK, JNK, and the p38 MAPK families of proteins are activated in response to neutrophil priming/activation (reviewed in [90]). It is also clear from previous studies that one or more of these MAPK pathways is induced by FPR agonists [91,92] and IL-8 [93]. Thus, these compounds and especially farnesene may be general inhibitors of neutrophil activation through GPCRs, and further studies are in progress to evaluate this idea and identify their specific molecular targets.

## 3. Materials and Methods

### 3.1. Materials

*N*-formyl-Met-Leu-Phe (*f*MLF), Trp-Lys-Tyr-Val-Met (WKYMVM), and farnesene (mixture of isomers) were from Sigma-Aldrich Chemical Co. (St. Louis, MO, USA). (±)-Bornyl acetate was from Cayman Chemical Company (Ann Arbor, MI, USA). Xanthoxylin was from TargetMol (Boston, MA, USA). Piperitone (mixture of isomers), (+)-camphor, and 1,8-cineole were from TCI America (Portland, OR, USA), and (−)-camphor was from Alfa Aesar (Ward Hill, MA, USA). Fluo-4AM was from Invitrogen (Carlsbad, CA, USA). Fetal bovine serum was from ATCC (Manassas, VA, USA). Hanks’ balanced salt solution without Ca^2+^ and Mg^2+^ (HBSS^–^) was from Life Technologies (Grand Island, NY, USA). We prepared HBSS^+^ by adding 1.3 mM CaCl_2_ and 1.0 mM MgSO_4_. Human interleukin-8 (IL-8) was purchased from Peprotech (Rocky Hill, NJ, USA).

### 3.2. Essential Oil Extraction

Essential oils were obtained by hydrodistillation of air-dried plant material. Hydrodistillation was performed with a Clevenger-type apparatus, as previously described [25]. To avoid artifacts, we used conditions accepted by the European Pharmacopoeia to avoid artifacts. Essential oil yields (*w*/*v*) were calculated based on the amount of air-dried plant material used. We prepared stock solutions of the essential oils in 10 mg/mL DMSO for biological evaluation. For gas-chromatographic (GC) analysis, samples were prepared in 10% *w*/*v n*-hexane.

### 3.3. GC-Flame Ionization Detector (GC-FID) and GC-Mass Spectrometry (GC-MS) Analysis

We performed GC-MS analysis using an Agilent 5975 GC-MSD system, as previously described [94]. An Agilent Innowax FSC column (60 m × 0.25 mm, 0.25 μm film thickness) was used with He as the carrier gas (0.8 mL/min). The GC oven temperature was kept at 60 °C for 10 min, increased to 220 °C at a rate of 4 °C/min, kept constant at 220 °C for 10 min, and then increased to 240 °C at a rate of 1 °C/min. The split ratio was adjusted to 40:1, and the injector temperature was 250 °C. MS spectra were monitored at 70 eV with a mass range of 35 to 450 *m*/*z*. GC analysis was carried out using an Agilent 6890 N GC system. To obtain the same elution order as with GC-MS, the line was split for FID and MS detectors, and a single injection was performed using the same column and appropriate operational conditions. The flame ionization detector (FID) temperature was 300 °C. The essential oil components were identified by co-injection with standards (whenever possible), which were purchased from commercial sources or isolated from natural sources. The identities of compounds were also using the MassFinder software 4.0 (Dr. Hochmuth Scientific Consulting, Hamburg, Germany), Adams Library, Wiley GC/MS Library (Wiley, Hoboken, NJ, USA), and NIST Library. Confirmation was also achieved using the in-house “Başer Library of Essential Oil Constituents” database. The database was created using chromatographic analysis of pure compounds run under identical conditions. Samples were spiked with a C_8_–C_40_ *n*-alkane standard solution (Fluka, Buchs, Switzerland) to determine relative retention indices (RRI). The FID chromatograms were used to calculate the relative amounts (%) of the separated compounds.

### 3.4. Isolation of Human Neutrophils

We obtained human neutrophils using blood collected from healthy donors. Blood collection was approved by the Institutional Review Board at Montana State University (Protocol #MQ041017). Neutrophils were isolated as described previously [95]. The isolated neutrophils were resuspended in HBSS^–^. Neutrophil preparations were routinely >95% pure and >98% viable, as determined by light microscopy and trypan blue exclusion, respectively.

### 3.5. Cell Culture

Human THP-1 monocytic cells obtained from ATCC (Manassas, VA, USA) were cultured in RPMI 1640 medium (Mediatech Inc., Herndon, VA, USA) supplemented with 10% (*v*/*v*) FBS, 100 μg/mL streptomycin, and 100 U/mL penicillin.

### 3.6. Ca^2+^ Mobilization Assay

Changes in intracellular Ca^2+^ concentrations ([Ca^2+^]_i_) were monitored with a FlexStation 3 (Molecular Devices, Sunnyvale, CA, USA). For these assays, neutrophils were loaded with 1.25 μg/mL Fluo-4AM and incubated in the dark at 37 °C for 30 min. The cells were then washed with HBSS^-^. The dye-loaded cells were resuspended in HBSS^+^ and pipetted into the wells of black microtiter plates at 2 × 10^5^ cells/well. To measure the direct effects of samples on [Ca^2+^]_i_, the test samples were added to the wells (final concentration of DMSO was 1%), and fluorescence was monitored (λ_ex_ = 485 nm, λ_em_ = 538 nm). Changes in fluorescence were monitored every 5 s at room temperature for 240 s. To evaluate the inhibitory effects of the test samples, the samples were added to the wells and incubated for 10 min, with the subsequent addition of 5 nM *f*MLF, 5 nM WKYMVM, or 25 nM IL-8. Responses were normalized to the response induced by control 5 nM *f*MLF, 5 nM WKYMVM, or 25 nM IL-8 alone without pretreatment. These responses were assigned as 100%. To calculate median effective concentrations (EC_50_ or IC_50_), we used curve fitting (at least five or six points) and nonlinear regression analysis of the dose–response curves. Curve fitting was performed with Prism 9 (GraphPad Software, Inc., San Diego, CA, USA).

### 3.7. Chemotaxis Assay

To evaluate effects of samples on neutrophil migration, we resuspended neutrophils in HBSS^+^ containing 2% (*v*/*v*) heat-inactivated FBS (2 × 10^6^ cells/mL). We analyzed chemotaxis using 96-well ChemoTx chambers (Neuroprobe, Gaithersburg, MD). The neutrophils were first preincubated with the indicated concentrations of test samples at room temperature for 30 min. The pretreated cells were then pipetted into the chamber upper wells (4 × 10^4^ cells/well). The lower wells contained 30 µL of HBSS^+^ with 2% (*v*/*v*) heat-inactivated FBS, the indicated test samples or control 1% DMSO, and 1 nM *f*MLF as the chemoattractant. Three lower wells were reserved for background controls (DMSO treated cells in the upper wells and DMSO without *f*MLF in the lower wells). We allowed the cells to migrate through the polycarbonate membrane filter for 60 min at 37 °C/5% CO_2_. We determined the number of migrated cells by measuring ATP in lysates of transmigrated cells and comparing this to a standard curve obtained with known neutrophil numbers, as described previously [23]. Calculation of median effective concentrations (IC_50_) was performed by nonlinear regression analysis of the dose-response curves.

### 3.8. Cytotoxicity Assay

We analyze cytotoxicity in human neutrophils or THP-1 monocytic cells. Cytotoxicity was analyzed using a CellTiter-Glo Luminescent Cell Viability Assay Kit (Promega, Madison, WI, USA). Briefly, the cells were incubated (10^4^ cells/well) with the indicated concentrations of essential oil or compound for 90 min (for neutrophils) or 24 h (for THP-1 cells) at 37 °C/5% CO_2_. After incubation, we added substrate. The samples were analyzed using a Fluoroscan Ascent FL microplate reader.

### 3.9. Molecular Modeling

The PharmMapper Server [96] was used to identify potential protein targets for (*E*,*E*)-α-farnesene (CID: 5281516) and (*Z*,*E*)-α-farnesene (CID: 5362889) (CIDs are compound identifiers in PubChem). PharmMapper recognizes potential targets based on “invert” pharmacophore mapping. The protein biotargets are represented by sets of pharmacophore points in reference databases incorporated in the software. We used PubChem (https://pubchem.ncbi.nlm.nih.gov; accessed on 20 February 2022) as a source of initial 3D structures for our compounds. These structures were downloaded from PubChem in SDF format. We then uploaded the structures into PharmMapper. The system automatically generated up to 300 conformers of each compound based on the software option. We performed pharmacophore mapping using the “Human Protein Targets Only” database, which contained 2241 targets. We retrieved the top 250 potential targets for each compound evaluated. The potential targets were sorted by normalized fit score. We calculated compound physicochemical properties using SwissADME (http://www.swissadme.ch; accessed on 20 March 2022). Properties were calculated for the structures of (−)-bornyl acetate (CID: 93009), (+)-bornyl acetate (CID: 6950274), (*E*,*E*)-α-farnesene (CID: 5281516), and (*Z*,*E*)-α-farnesene (CID: 5362889). The alignment of lipoxin-A_4_, (*E*,*E*)-α-farnesene, and (*Z*,*E*)-α-farnesene was made with the use of FieldTemplater software (Cresset Group, England, UK).

### 3.10. Statistical Analysis

For statistical analysis, we performed a one-way analysis of variance (ANOVA), followed by Tukey's pair-wise comparisons. We considered differences at *p* < 0.05 to be statistically significant.

## 4. Conclusions

Our results demonstrate that: (1) *Artemisia* spp. essential oils contain compounds that exhibit neutrophil immunomodulatory activity, which might contribute to the reported pharmacological properties of extracts from these plants; (2) the biological effects of these compounds on neutrophil function are not due to compound cytotoxicity; (3) essential oils from *A. dracunculus* flowers (AD_Fl_) contain farnesene isomers that may have anti-inflammatory activity due to their ability to inhibit neutrophil responses to inflammatory chemoattractants, establishing farnesene as a novel innate immunomodulator, and (4) synergetic effects may be possible with other essential oil constituents, such as bornyl acetate or xanthoxylin. Further work is in progress to define the mechanisms of farnesene action and evaluate its therapeutic potential.

## Figures and Tables

**Table 1:** Location and date of collection of the plant material.

*Artemisia* spp.	Location	Latitude (N)	Longitude (E)	Altitude (m)	Plant Material	Date of Collection; Specimen No.	Yield (%)
*A. ludoviciana*	Three Forks, MT	45.92721	111.50106	1235	leaves/flowers	August 2019; 2019-IAS-A1	0.3/1.9
*A. dracunculus*	Bozeman, MT	45.71475°	110.97890°	1646	leaves/flowers	August 2019; 2019-IAS-A2	0.9/0.9
*A. frigida*	Three Forks, MT	45.92553°	111.49730°	1240	leaves/flowers	August 2019; 2019-IAS-A3	0.3/1.9
*A. cana*	Three Forks, MT	45.92274°	111.49453°	1238	leaves/flowers	August 2019; 2019-IAS-A4	1.4/1.5
*A. tridentata*	Bozeman, MT	45.74118°	110.98698°	1415	leaves/flowers	August 2019; 2019-IAS-A5	3.3/2.7

**Table 2:** Composition of essential oils (%) isolated from leaves and flowers of five *Artemisia* species.

No	RRI^a^	RRI^b^	Compound	AT_Lv_	AT_Fl_	AL_Lv_	AL_Fl_	AD_Lv_	AD_Fl_	AF_Lv_	AF_Fl_	AC_Lv_	AC_Fl_
1	1032	1008–1039 ^#^	α-Pinene	2.1	1.8	2.5	3.9	0.1	0.1	2.2	3.3	0.8	2.0
2	1043	1011–1063 ^#^	Santolinatriene	0.7	1.7		3.5					2.2	2.4
3	1076	1043–1086 ^#^	Camphene	7.7	7.7	7.1	5.5	t	t	4.0	6.0	1.6	4.1
4	1189	1179 ^@^	Artemiseole	2.1	3.1		2.5					7.5	1.2
5	1213	1186–1231 ^#^	1,8-Cineole	21.8	23.8	16.3	23.1			12.5	5.7	30.0	21.9
6	1218	1188–1233 ^#^	β-Phellandrene					1.3	2.2				
7	1246	1211–1251 ^#^	(*Z*)-β-Ocimene					4.6	9.4				
8	1266	1232–1267 ^#^	(*E*)-β-Ocimene					2.7	6.6	t			
9	1280	1246–1291 ^#^	*p*-Cymene	1.2	1.2	0.7	0.7	0.5	0.2	3.2	2.1	0.9	0.7
10	1290	1260–1300 ^#^	Terpinolene	t	t	0.2	0.3	8.8	5.5	0.1	0.3	0.1	0.1
11	1329	1312 ^	Santolina epoxide *										3.1
12	1451	1400–1452 ^#^	β-Thujone							3.3	0.1		
13	1474	1453 **	*trans*-Chrysanthenol							2.0			
14	1532	1481–1537 ^#^	Camphor	51.3	41.7	41.1	26.6			23.0	37.7	32.5	35.9
15	1538	1533–1590 ^#^	*trans*-Chrysanthenyl acetate							8.1			
16	1553	1507–1564 ^#^	Linalool			3.8	2.5	0.1	0.2				
17	1590	1549–1597 ^#^	Bornyl acetate	0.9	0.6	0.3	0.6			1.5	3.8	0.3	0.8
18	1611	1564–1630 ^#^	Terpinen-4-ol	2.7	2.9	2.4	3.8		t	3.3	6.9	2.0	2.3
19	1687	1652–1690 ^#^	Methyl chavicol					42.9	38.8				
20	1719	1653–1728 ^#^	Borneol	2.4	3.3	9.6	8.1		t	6.6	14.6	2.9	2.3
21	1737	1713–1748 ^#^	(*Z,E*)-α-Farnesene						t				
22	1748	1689–1748 ^#^	Piperitone				0.2			1.5	1.1		
23	1758	1714–1763 ^#^	(*E,E*)-α-Farnesene					0.2	0.5				
24	1764	1751–1765 ^#^	*cis*-Chrysanthenol	0.5	0.8	1	0.5			3.9	0.5		0.2
25	1821	1807 ***	Fragranol									3.0	
26	1827	1827 ***	Grandisol										3.9
27	1864	1813–1865 ^#^	*p*-Cymen-8-ol	0.2	0.1	0.1		2.3	0.9	0.4	0.3	0.1	0.1
28	2030	1961–2033 ^#^	Methyl eugenol					26.1	26.4	t	0.1		
29	2637	2608 ^##^	Xanthoxylin					2.2	1.2				
				**Summary of the composition of *Artemisia* spp. essential oils**									
Compounds				AT_Lv_	AT_Fl_	AL_Lv_	AL_Fl_	AD_Lv_	AD_Fl_	AF_Lv_	AF_Fl_	AC_Lv_	AC_Fl_
Monoterpene Hydrocarbons				13.1	14.4	13.5	18.7	20.3	25.8	11.6	17.4	6.5	11.3
Oxygenated Monoterpenes				84.5	79.8	82.5	77.4	4.0	1.9	79.6	79.8	84.8	81.0
Sesquiterpene Hydrocarbons					2.0	0.2	0.7	1.7	2.4	0.6	0.2	0.1	0.3
Oxygenated Sesquiterpenes				0.8	1.6	1.1	1.2	1.7	1.1	2.5	0.6	0.1	0.4
Phenylpropanoids						1.3	0.2	69.0	65.3		0.1		0.1
Others				t	t	0.4	0.2	2.6	2.1	0.2	0.5	t	0.6
Total				98.0	97.1	98.9	97.8	98.2	97.7	94.5	98.4	91.5	93.6

**Legend:** The data are presented as relative % for each component identified. RRI^a^, relative retention index calculated based on retention of *n*-alkanes. RRI^b^, relative retention indexes reported in the literature: ^#^ [28], ^@^ [26]; ^ [29]; ** [30], *** [31,32], ^##^ [33]. % was calculated from flame ionization detector data. Trace amounts (t) were present at <0.1%. * Santolina epoxide was tentatively identified using Wiley and MassFinder mass spectra libraries and published RRI. All other compounds were identified by comparison with co-injected standards. Abbreviations for the oil samples: AT_Lv_, *A. tridentata* leaves; AT_Fl_, *A. tridentata* flowers; AL_Lv_, *A. ludoviciana* leaves; AL_Fl_, *A. ludoviciana* flowers; AD_Lv_, *A. dracunculus* leaves; AD_Fl_, *A. dracunculus* flowers; AF_Lv_, *A. frigida* leaves; AF_Fl_, *A. frigida* flowers; AC_Lv_, *A. cana* leaves; AC_Fl_, *A. cana* flowers.

**Table 3:** Effect of essential oils from *Artemisia* spp. and pure component compounds on [Ca^2+^]_i_ and cytotoxicity in human neutrophils.

Essential Oil or Pure Compound	Activation of [Ca^2+^]_i_	Inhibition of [Ca^2+^]_i_		Cytotoxicity
		*f*MLF-Induced	WKYMVM-Induced	
	EC_50_ (μg/mL)	IC_50_ (μg/mL)		IC_50_ (μg/mL)
AT_Lv_	23.6 ± 3.6	28.9 ± 4.1	3.4 ± 0.8	Nontoxic
AT_Fl_	32.1 ± 4.5	19.5 ± 3.3	3.7 ± 0.1	Nontoxic
AL_Lv_	15.8 ± 2.1	31.2 ± 3.9	8.1 ± 3.4	Nontoxic
AL_Fl_	16.3 ± 4.2	21.4 ± 3.2	19.6 ± 6.3	Nontoxic
AD_Lv_	25.3 ± 7.4	4.6 ± 1.3	5.3 ± 1.8	32.6 ± 4.1
AD_Fl_	18.8 ± 3.2	2.2 ± 0.8	2.9 ± 0.8	24.7 ± 2.8
AF_Lv_	44.7 ± 6.2	19.3 ± 4.2	32.4 ± 6.4	42.4 ± 3.2
AF_Fl_	41.5 ± 3.5	13.0 ± 2.6	26.6 ± 6.5	31.4 ± 4.8
AC_Lv_	48.7 ± 9.5	18.4 ± 7.6	36.5 ± 4.8	Nontoxic
AC_Fl_	41.6 ± 11.7	22.6 ± 6.8	30.4 ± 7.1	Nontoxic
	**EC_50_ (μM)**	**IC_50_ (μM)**	**IC_50_ (μM)**	
1,8-Cineole	N.A.	N.A.	N.A.	Nontoxic
(−)-Camphor	N.A.	N.A.	N.A.	Nontoxic
(+)-Camphor	N.A.	N.A.	N.A.	Nontoxic
(±)-Bornyl acetate	50.1 ± 11.5	42.6 ± 9.7	19.1 ± 0.1	Nontoxic
Farnesene	28.5 ± 2.6	1.1 ± 0.2	1.4 ± 0.5	Nontoxic
Piperitone	N.A.	N.A.	N.A.	Nontoxic
Xanthoxylin	53.3 ± 5.0	27.2 ± 6.6	52.7 ± 11.2	Nontoxic

**Legend:** EC_50_ and IC_50_ values were determined by nonlinear regression analysis of the dose-response curves. For the determination of cytotoxicity, neutrophils were incubated with indicated concentrations of the compounds for 90 min and cell viability was analyzed. N.A. indicates the samples had essentially no activity or no cytotoxicity, respectively (EC_50_ or IC_50_ > 55 µM for pure compounds or > 55 µg/mL for the oils). Presented as the mean ± SD of three independent experiments. Abbreviations for the oils: AT_Lv_, *A. tridentata* leaves; AT_Fl_, *A. tridentata* flowers; AL_Lv_, *A. ludoviciana* leaves; AL_Fl_, *A. ludoviciana* flowers; AD_Lv_, *A. dracunculus* leaves; AD_Fl_, *A. dracunculus* flowers; AF_Lv_, *A. frigida* leaves; AF_Fl_, *A. frigida* flowers; AC_Lv_, *A. cana* leaves; AC_Fl_, *A. cana* flowers.

**Table 4:** Predicted physicochemical properties of farnesene isomers and bornyl acetate.

Property	(*E*,*E*)-α-Farnesene	(*Z*,*E*)-α-Farnesene	Bornyl Acetate
Formula	C_15_H_24_	C_15_H_24_	C_12_H_20_O_2_
M.W.	204.35	204.35	196.29
Heavy Atoms	15	15	14
Fraction Csp^3^	0.47	0.47	0.92
Rotatable Bonds	6	6	2
H-Bond Acceptors	0	0	2
H-Bond Donors	0	0	0
MR	72.32	72.32	56.33
tPSA	0.00	0.00	26.30
LogP	5.70	5.70	3.50
BBB Permeation	Yes	Yes	Yes

**Legend**: M.W., molecular weight (g/mol); MR, molar refractivity; tPSA, topological polar surface area (Å^2^); LogP, lipophilicity; BBB, blood–brain barrier.

**Table 5:** Potential protein targets for bornyl acetate and farnesene were identified by PharmMapper.

Rank	PDB ID	Target Name	Fit Score	Rank	PDB ID	Target Name	Fit Score
(−)-Bornyl Acetate				(+)-Bornyl Acetate			
1	1REU	BMP2	1	1	1J96	AKR1C2	1
2	1J96	AKR1C2	1	2	1REU	BMP2	1
3	1MX1	LCE1	0.996	3	1OKL	CA2	0.9992
4	2AO6	NR3C4	0.9925	4	2PIQ	NR3C4	0.9962
5	2PE0	PDPK1	0.9923	5	2G01	JNK1	0.9857
6	2G01	JNK1	0.9848	6	1W8L	PPIase A	0.9826
7	1IF4	CA2	0.9815	7	2UZD	Cyclin-A2	0.9821
8	1W8L	PPIase A	0.9811	8	1MX1	LCE1	0.9784
9	1VJY	TGFBR1	0.979	9	1A28	PgR	0.9771
10	1A28	PgR	0.9636	10	2PE0	PDPK1	0.9711
**(*E*,*E*)-α-Farnesene**				**(*Z*,*E*)-α-Farnesene**			
1	1J96	AKR1C2	1	1	1PME	JNK1	1
2	3HVC	p38α	1	2	3HVC	p38α	1
3	1E7A	Serum albumin	1	3	3BGP	Pim-1	0.9998
4	1OJ9	MAO-B	1	4	2PG2	KIF11	0.9993
5	1SHJ	Caspase-7	1	5	1E7A	Serum albumin	0.999
6	1PME	ERK2	1	6	1OJ9	MAO-B	0.9989
7	1P49	Steryl-sulfatase	0.9989	7	1J96	AKR1C2	0.9988
8	2PIN	NR1A2	0.9985	8	1L6L	Apo A-II	0.9984
9	3BGP	Pim-1	0.9982	9	2PIN	NR1A2	0.9978
10	1L6L	Apo A-II	0.9977	10	1P49	Steryl-sulfatase	0.9975

**Legend**: AKR1C2, aldo-keto reductase family 1 member C2; Apo A-II, apolipoprotein A-II; BMP2, bone morphogenetic protein 2; CA2, carbonic anhydrase 2; ERK2, extracellular signal-regulated kinase 2; JNK1, c-Jun N-terminal kinase 1; KIF11, kinesin family member 11; LCE1, liver carboxylesterase 1; MAO-B, monoamine oxidase B; NR1A2, thyroid hormone receptor β; NR3C4, androgen receptor; p38α, p38α mitogen-activated protein kinase; PDPK1, 3-phosphoinositide-dependent protein kinase 1; PgR, progesterone receptor; Pim-1, proto-oncogene serine/threonine-protein kinase; PPIase A, peptidyl-prolyl *cis*-trans isomerase A; TGFBR1, TGF-β receptor type-1.

## Data Availability

Data are contained within the article and supplementary material.

## Supplementary Materials

The following supporting information can be downloaded at: https://www.mdpi.com/article/10.3390/ph15050642/s1, Table S1: includes all compounds found in the essential oils from each *Artemisia* species.
