# Supplementary material for: Neutrophil Immunomodulatory Activity of Farnesene, a Component of Artemisia dracunculus Essential Oils
Source: Pharmaceuticals (Basel). 2022 May 23;15(5):642. doi: 10.3390/ph15050642 (PMC9143003; doi:10.3390/ph15050642)
Supplement: Supplementary file 1 [file pharmaceuticals-15-00642-s001.zip › pharmaceuticals-1715781-supplementary.pdf]

Supplementary Material

for

Neutrophil Immunomodulatory Activity of Farnesene, a Component of *Artemisia dracunculus* Essential Oils

by

Igor A. Schepetkin[1], Gulmira Özek[2], Temel Özek[2], Liliya N. Kirpotina[1], Andrei I. Khlebnikov[3], Robyn A. Klein[4], and Mark T. Quinn[1]

[1]*Department of Microbiology and Cell Biology, Montana State University, Bozeman, MT 59717, United States*

[2]*Department of Pharmacognosy, Faculty of Pharmacy, Anadolu University, Eskisehir 26470, Turkey*

[3]*Kizhner Research Center, Tomsk Polytechnic University, Tomsk, Russia*

[4]*Department of Plant Sciences and Plant Pathology, Montana State University, Bozeman, MT 59717, United States*

Supplemental Table S1. Chemical composition of essential oils (%) isolated from leaves and flowers of different Artemisia species.

No	RRI	Compound	AT_{Lv}	AT_{Fl}	AL_{Lv}	AL_{Fl}	AD_{Lv}	AD_{Fl}	AF_{Lv}	AF_{Fl}	AC_{Lv}	AC_{Fl}
1	956	1,3-Octadiene					0.2	0.3				
2	1014	Tricyclene	0.3	0.3	0.3	0.3			0.2	0.3	0.1	0.2
3	1018	Methyl 2-methyl-butyrate						t				
4	1024	2-Butanol					0.1	0.1	0.1			
5	1032	α-Pinene	2.1	1.8	2.5	3.9	0.1	0.1	2.2	3.3	0.8	2.0
6	1035	α-Thujene	t		t	0.5	t	t	t	0.1		t
7	1043	Santolinatriene	0.7	1.7		3.5					2.2	2.4
8	1048	2-Methyl-3-buten-2-ol					t	0.1				
9	1072	α-Fenchene					0.1	0.1				
10	1076	Camphene	7.7	7.7	7.1	5.5	t	t	4.0	6.0	1.6	4.1
11	1093	Hexanal						t				t
12	1094	2-Methyl-2-butenal						t				
13	1115	(*E*,*Z*)-1,3,5-Octatriene						t				
14	1118	β-Pinene	0.7	0.9	0.9	1.4	t	t	0.6	1.4	0.4	1
15	1129	Thuja-2,4(10)-diene				0.1			t			t
16	1132	Sabinene	t	t	0.3	0.7	t	0.1	0.3	0.3		0.1
17	1159	δ-3-Carene					0.1	t				
18	1174	Myrcene		t		0.1	0.6	0.3	0.1	1.3		
19	1176	α-Phellandrene	t	t		0.3	0.4	0.3	t	t		t
20	1188	α-Terpinene			0.6		0.1	0.1	0.2	0.6		
21	1189	Artemisiole	2.1	3.1		2.5					7.5	1.2
22	1195	Dehydro-1,8-cineole	t	t	0.2	0.1	t	t	0.1	t	0.2	0.1
23	1203	2-Methyl butyl isobutyrate				t			t			
24	1203	Limonene	0.2	0.3	0.2	0.2	0.5	0.5	0.3	0.4		0.2
25	1213	1,8-Cineole	21.8	23.8	16.3	23.1			12.5	5.7	30	21.9
26	1218	β-Phellandrene					1.3	2.2				
27	1224	*o*-Mentha-1(7),5,8-triene		t								
28	1225	(*Z*)-3-Hexenal		t	t		t	0.1	t	t	t	t
29	1244	Amyl furan		t	t				t			t
30	1246	(*Z*)-β-Ocimene					4.6	9.4				
31	1253	*trans*-Anhydrolinalool oxide	t	t	t							
32	1255	γ-Terpinene	0.2	0.5	0.7	1.2	0.1	t		1.3	0.4	0.5
33	1266	(*E*)-β-Ocimene					2.7	6.6	t			
34	1268	Prenyl acetate										0.1
35	1280	*p*-Cymene	1.2	1.2	0.7	0.7	0.5	0.2	3.2	2.1	0.9	0.7
36	1290	Terpinolene	t	t	0.2	0.3	8.8	5.5	0.1	0.3	0.1	0.1
37	1294	1,2,4-Trimethyl benzene							t			
38	1296	Octanal								t		t
39	1319	(*E*)-2,6-Dimethyl-1,3,7-nonatriene										0.2
40	1324	Prenyl isobutyrate										0.2
41	1327	3-Methyl-2-butenol										t
42	1329	Santolina epoxide*										3.1
43	1327	(*Z*)-3-hexenyl acetate						0.1				
44	1348	6-Methyl-5-hepten-2-one						t				
45	1342	Santolina epoxide isomer*										0.3
46	1360	Hexanol										t
47	1379	3-Methyl-3-butenyl isovalerate										t
48	1382	*cis*-Alloocimene					0.2	0.3				
49	1384	α-Pinene oxide					0.1	0.1				
50	1400	Nonanal								0.1		
51	1403	Yomogi alcohol				1.7					0.5	0.3
52	1405	Santolina alcohol									0.3	
53	1408	1,3,8-*p*-Menthatriene					t					

54	1413	Rose furan					t	t				
55	1414	Presilphiperfol-7-ene					0.1	t				
56	1432	7-α-(*H*)-silphiperfol-5-ene					t					
57	1436	*p*-Menthatriene isomer					t	t				
58	1437	α-Thujone							0.2			
59	1443	2,5- Dimethylstyrene					0.2	0.1	0.1	t		
60	1445	Filifolone	0.1	t					0.1			
61	1446	2,6-Dimethyl-1,3(*E*),5(*Z*),7-octatetraene					t	t				
62	1451	β-Thujone							3.3	0.1		
63	1452	α,*p*-Dimethylstyrene	t	t								
64	1452	1-Octen-3-ol			0.4	0.2						0.1
65	1458	*cis*-1,2-Limonene epoxide										t
66	1460	7-β-(*H*)-Silphiperfol-5-ene					0.1	t				
67	1465	Eucarvone							0.1			
68	1474	Camphenilone							0.1	0.1	0.1	0.1
69	1474	trans-Chrysanthenol							2			
70	1474	*trans*-Sabinene hydrate			0.6				0.3	1.1	0.3	0.7
71	1476	(*Z*)-β-Ocimene epoxide					t	t			0.1	
72	1477	4,8-Epoxyterpinolene					0.6	0.2				
73	1478	*cis*-Linalool oxide			t							
74	1480	Nerol oxide	t	t								t
75	1482	Siphin-1-ene						t				
76	1497	α-Copaene		t	0.2		t	0.1		0.2		0.2
77	1498	(*E*)-β-Ocimene epoxide					t	0.1			0.1	
78	1499	Silphiperfol-6-ene					0.2	0.1				
79	1499	α-Campholene aldehyde										0.3
80	1510	Artemisia alcohol				0.8					0.7	0.2
81	1522	Chrysanthenone							1			
82	1526	(Z)-2,6-Dimethylocta-1,5,7-trien-3-ol*										0.1
83	1532	Camphor	51.3	41.7	41.1	26.6			23.0	37.7	32.5	35.9
84	1535	β-Bourbonene						t				
85	1534	1-Nonen-3-ol										t
86	1538	*trans*-Chrysanthenyl acetate							8.1			
87	1547	Dihydroachillene							0.3			
88	1553	Linalool			3.8	2.5	0.1	0.2				
89	1556	*cis*-Sabinene hydrate			0.5				0.3	1.4	0.5	0.8
90	1558	(*E*)-2,6-Dimethylocta-1,5,7-trien-3-ol*										0.3
91	1571	*trans*-*p*-Menth-2-en-1-ol			0.3	0.3	t	t	0.8	0.9	0.4	0.5
92	1582	*cis*-Chrysanthenyl acetate	t		0.2				1	0.1		
93	1586	Pinocarvone	0.3	0.4	0.6	0.4			1.6	1.2	0.5	0.7
94	1589	β-Ylangene						t				
95	1590	Bornyl acetate	0.9	0.6	0.3	0.6			1.5	3.8	0.3	0.8
96	1608	Nopinone	0.2	0.3						0.1		
97	1609	Camphene hydrate	t									
98	1611	Terpinen-4-ol	2.7	2.9	2.4	3.8		t	3.3	6.9	2	2.3
99	1612	β-Caryophyllene	t	0.8			0.1	0.1				
100	1616	Hotrienol			0.6	0.4						
101	1617	Lavandulyl acetate							0.3	t		
102	1624	*trans*-Dihydrocarvone	0.1	0.2								
103	1630	Terpinen-4-yl acetate							0.1	0.1		t
104	1638	*cis*-*p*-Menth-2-en-1-ol			0.1	0.2	t	t	0.4	0.5	0.2	0.1
105	1639	*trans*-*p*-Mentha-2,8-dien-1-ol		t								
106	1645	*cis*-Verbenyl acetate										0.1
107	1645	*cis*-Isodihydrocarvone										t
108	1648	Myrtenal			0.1				0.1	0.2		
109	1658	Sabinyl acetate							1.3			
110	1663	*cis*-Verbenol			0.1							

111	1668	Citronellyl acetate					0.1	t				
112	1670	*trans*-Pinocarveol	0.6	0.7	0.5	0.3			0.6	0.6	0.4	0.3
113	1678	*cis-p*-Mentha-2,8-dien-1-ol	t									
114	1681	(*Z*)-3-Hexenyl tiglate						t				
115	1682	δ-Terpineol	0.3	0.3	0.4	0.4			0.2		0.3	0.3
116	1683	*trans*-Verbenol			0.5				0.2		0.3	0.4
117	1684	Chrysanthenyl isovalerate I							0.1			
118	1684	*trans*-Chrysanthemol				0.8						0.1
119	1686	Lavandulol							0.1	0.3		
120	1687	Methyl chavicol					42.9	38.8				
121	1688	Selina-4,11-diene								t		
122	1689	*trans*-Piperitol				t			0.2	0.4		
123	1700	*p*-Mentha-1,8-dien-4-ol					0.2	0.1		0.1		
124	1704	γ-Curcumene		0.3		t	0.1	0.2				
125	1706	α-Terpineol	0.8	1.3	1.3	1.7			0.3	0.4	0.8	0.8
126	1719	Borneol	2.4	3.3	9.6	8.1		t	6.6	14.6	2.9	2.3
127	1719	Fragranyl acetate									t	2
128	1720	*trans*-Sabinol							1.7			
129	1725	Verbenone				1.3				t		
130	1726	Germacrene D		0.6	t	0.2	0.7	1	0.5		0.1	0.1
131	1740	Geranial							0.1			
132	1737	(*Z,E*)-α-Farnesene						t				
133	1738	*p*-Mentha-1,5-dien-8-ol	t	t	0.1	0.3		t			t	
134	1747	*trans*-Carvyl acetate	t		0.2					0.1		t
135	1748	Piperitone				0.2			1.5	1.1		
136	1751	Carvone									t	
137	1755	Bicyclogermacrene						0.1				
138	1758	*cis*-Piperitol									0.1	0.1
139	1758	(*E,E*)-α-Farnesene					0.2	0.5				
140	1764	*cis*-Chrysanthenol	0.5	0.8	1	0.5			3.9	0.5		0.2
141	1772	Citronellol					0.3	0.1				
142	1773	δ-Cadinene						0.1				t
143	1776	γ-Cadinene						t				
144	1782	*cis*-Carvyl acetate			0.2							
145	1786	*ar*-Curcumene		0.3		0.5	0.2	0.2				
146	1797	*p*-Methyl acetophenone					0.1	0.1				
147	1798	Methyl salicylate			t			t				
148	1802	Cumin aldehyde							0.2			
149	1804	Myrtenol		0.2	0.2	0.2			0.1	0.2	0.2	0.2
150	1805	α-Campholene alcohol				0.2				0.1	0.2	0.2
151	1807	Perilla aldehyde										t
152	1811	*trans-p*-Mentha-1(7),8-dien-2-ol								0.2	t	
153	1821	Fragranol									3	
154	1827	(*E,E*)-2,4-Decadienal						t				
155	1827	Grandisol										3.9
156	1830	2,6-Dimethyl-3(*E*),5(*E*),7-octatriene-2-ol					0.2	0.2				
157	1845	(*E*)-Anethole						t				
158	1845	*trans*-Carveol	0.2	0.1	0.7	0.1			0.2	0.1	0.1	0.1
159	1853	*cis*-Calamenene						t	0.1			
160	1864	*p*-Cymen-8-ol	0.2	0.1	0.1		2.3	0.9	0.4	0.3	0.1	0.1
161	1865	Isopiperitenone							t			
162	1870	Hexanoic acid							0.1	0.3		t
163	1882	*cis*-Carveol			0.2	0.2						
164	1889	Ascaridole								0.1	0.1	
165	1896	*cis-p*-Mentha-1(7),8-diene-2-ol								0.1		0.1
166	1900	Nonadecane						t				
167	1900	*epi*-Cubebol						t				

168	1969	*cis*-Jasmone			0.3	0.1		t	0.2	0.1	t	t
169	1992	Cameroonan-7-α-ol					0.1	t				
170	2006	Davanone							t			
171	2008	Caryophyllene oxide		0.1	0.1			t	0.1	0.1		
172	2008	Junenol										t
173	2029	Perilla alcohol									0.1	0.1
174	2030	Methyl eugenol					26.1	26.4	t	0.1		
175	2032	Artedouglasia oxide I							0.4			
176	2037	Salvial-4(14)-en-1-one			t					t		t
177	2045	Davanone isomer							0.1			
178	2049	*cis*-Davanone	0.4	0.6		0.2			1			
179	2050	(*E*)-Nerolidol				0.3	0.2	0.1				
180	2057	Ledol										t
181	2069	1,6-Germacradien-5β-ol						t				
182	2071	Presilphiperfolan-8-ol					0.4	0.2				
183	2073	*p*-Mentha-1,4-dien-7-ol							t			
184	2074	3-exo-Acetoxyborneol*							0.1			
185	2088	1-*epi*-Cubenol							t			
186	2092	Artedouglasia oxide II							0.1			
187	2100	Heneicosane						0.1				
188	2113	Cumin alcohol							0.3	0.1		
189	2144	Spathulenol		0.1	0.1		0.2	0.2	0.1	t		0.1
190	2162	Fokienol			t	0.1						
191	2170	β-Bisabolol						t				
192	2174	Cinnamyl acetate						t				
193	2181	Isothymol								0.1		
194	2186	Eugenol			1.3	0.2		0.1				0.1
195	2187	τ-Cadinol		0.1								
196	2192	Nonanoic acid								0.1		
197	2198	Thymol							0.1	0.3		
198	2209	τ-Muurolol			0.2	t		t				
199	2217	α-Cedrenal								0.2		
200	2221	Isocarvacrol							0.7			
201	2225	Copaborneol			t				0.3	0.1		
202	2239	Carvacrol							0.3	0.1		
203	2250	Torilenol						t				0.1
204	2254	β-Copaen-4 a-ol*										0.1
205	2255	α-Cadinol						t				
206	2257	β-Eudesmol			0.2							
207	2273	Selin-11-en-4α-ol							0.4			
208	2300	Tricosane						t				
209	2324	Caryophylla-2(12),6(13)-dien-5α-ol		t								
210	2368	Eudesma-4(15),7-diene-1-β-ol										t
211	2392	Caryophylla-2(12),6-dien-5β-ol			0.2						0.1	
212	2430	Chamazulene			0.2							
213	2637	Xanthoxylin *					2.2	1.2				

Abbreviations: AT_{Lv}, *A. tridentate* leaves; AT_{Fl}, *A. tridentate* flowers; AL_{Lv}, *A. ludovicana* leaves; AL_{Fl}, *A. ludovicana* flowers; AD_{Lv}, *A. dracunculus* leaves; AD_{Fl}, *A. dracunculus* flowers; AF_{Lv}, *A. frigida* leaves; AF_{Fl}, *A. frigida* flowers; AC_{Lv}, *A. cana* leaves; AC_{Fl}, *A. cana* flowers. *Tentatively identified using Wiley and MassFinder mass spectra libraries and published RRI. All other compounds were identified by comparison with coinjected standards.
